# Correction: Novakovskaya et al. The Fate of Secondary Electrons in Water upon High-Energy Electron Impact: Changes in the Presence of Nanobubbles. *Int. J. Mol. Sci.* 2025, *26,* 8909

**DOI:** 10.3390/ijms262311343

**Published:** 2025-11-24

**Authors:** Yulia V. Novakovskaya, Nikolai F. Bunkin, Sergey A. Tarasov, Natalia N. Rodionova, Anastasia O. Petrova, German O. Stepanov

**Affiliations:** 1 Chemistry Department, Lomonosov Moscow State University, Leninskie Gory, 1/3, 119991 Moscow, Russia; 2 Department of Fundamental Sciences, Bauman Moscow State Technical University, 2nd Baumanskaya Str. 5, 105005 Moscow, Russia; 3 Research and Development Department, OOO “NPF “Materia Medica Holding”, 129272 Moscow, Russia or satarasovmail@yandex.ru (S.A.T.); rodionovann@materiamedica.ru (N.N.R.); petrovaao@materiamedica.ru (A.O.P.); or stepanov_go@rsmu.ru (G.O.S.); 4 Department of Molecular and Cellular Pathophysiology, Institute of General Pathology and Pathophysiology, 125315 Moscow, Russia; 5 Institute of Biomedicine, Pirogov Russian National Research Medical University, Ministry of Health of the Russian Federation, 117513 Moscow, Russia

In the original publication [1], there was a mistake in the column titles of Table 4. The units of all energy parameters should be kcal/mol. In the discussion, correct values are quoted. The corrected Table 4 appears below. The authors state that the scientific conclusions are unaffected. This correction was approved by the Academic Editor. The original publication has also been updated.

## Figures and Tables

**Table 4:** Structural and energetic characteristics of the excess electron localization in the [(H_2_O)_27_]^−^ cluster: coordination number of the electron (*CN*), the character of its localization, the relative energy (*E_rel_*) and vibrational Gibbs energy (*G_vib_*) of the cluster, the cluster reorganization energy (*E_reorg_*), and the vertical (*VDE*) and adiabatic (*ADE*) electron detachment energies (energies in kcal/mol).

CN	Localization Kind	*E_rel_*	*G_vib_*	*E_reorg_*	*VDE*	*ADE*
3	Subsurface	3.9	4.8	39.1	22.6	−16.5
4	Surface	0.0	0.0	41.1	35.3	−5.9
6	Bulk	5.8	4.5	58.9	62.7	3.8
